# A Brief History of an Existing Controversy on the Subject of Assimilated Rank in the Navy of the United States

**Published:** 1850-11

**Authors:** 


"*A brief history of an existing controversy on the subject of Assimilated Rank in the Navy of the United States. By* W. S. W. R. Philadelphia, C. Sherman, Printer, 1850.

This pamphlet, as its title imports, is a summary and exposition of several publications which have lately appeared in this city and elsewhere, on the question of apportioning rank, *so far as it relates to official etiquette*, throughout the naval service; a subject, strange to say, hitherto undetermined by Congressional enactment.

If the author really considers the pamphlet "brief," his facility in the labors of composition and penmanship are greatly to be envied; but any one who will take the trouble to read it carefully through, will find that it exposes a degree of monomaniacal jealousy and distrust, among a large body of naval officers, which it is not easy to credit, much less explain.

The case stands thus. The Post Captains, Commanders and Lieutenants of the Navy wish to exclude the Surgeons and Pursers from all the ceremonial honors which (*apart from command, be it understood*) define and secure military rank. So entirely hostile is the spirit of this republican government to all such exclusive organizations of mere arbitrary, irrelavent power against scholastic furniture and financial responsibility, that the time has arrived when these gentlemen have become doubtful and anxious about the fairness and stability of these, their ancient usages, and are endeavoring to perpetuate their absurd claims by narrow and illogical appeals to the Navy Department and Congress.

Their first argument is based upon what they consider the merits of inviolate custom, and, with this huge, two-handed sword, they expect to ward off the ameliorating statutes of a higher civilization. Alas for ancient usage! how unceremoniously has the lash been taken from their grasp, and how significantly has the slumbering genius of their better nature been invoked to find some means of rule less repugnant to their sober selves—less shame-effacing to their brother sailor!

The further argument of these gentlemen is, that Surgeons and Pursers wish to command them. How utterly preposterous and untrue this is, every naval officer, blessed with a mind and unscathed by a reckless dissipation, must be brought to admit. It is *assimilated and not executive rank* that Surgeons and Pursers ask for. So far from having a thought of command, *they repudiate the idea in its any and every sense.* Their education, their habits, their occupations are all at variance with such an exercise of their human faculties. *Their aspirations are infinitely above it:* it would be incompatible, uncongenial, irrelavent. The mission of the one is to the sick and suffering—the ever various duties of the other demand the closest official fidelity. Where are the qualifications for *command* here? Where is the Surgeon who would willingly disparage the high honor of his art by the exercise of mere military authority? Where the Purser who would care to confound his calculations by the petty vexations of command? No:

all *that* is left where it belongs. Let these combatant gentlemen carry their shipmates safely into port, and triumphantly through battle, and they, in return, will confine themselves strictly to *their* professions, and, in so doing, practically refute all such imputations.

This subject invites reference to another pamphlet, recently addressed to the Hon. Secretary of the Navy, and signed by twenty-four post captains, forty-two commanders, and seventy-six lieutenants. Is this the great gun of these gentlemen—the Peace Maker? Let us examine it. Upon looking over this formidable party list, we find that some, probably many, put their names to it, either without being at all aware of the character of the document they were called on to sign, or else because it was said to them—"These doctors and pursers wish to deprive you of command, and nothing but wide concert and extreme measures can defeat their ambitious designs." This is made apparent by the fact that some of these gentlemen have privately expressed their opinion in favor of the justice and expediency of assigning assimilated rank to Surgeons and Pursers, so that the only question remaining with them would seem to be regarding the precise definition of that rank. Then how strangely and mischievously have they contradicted themselves on the title page of this pamphlet, which aims at the total defeat, without qualification, of all assimilated rank with their brother officers! The title runs thus—"Assimilated Rank: its injurious operation upon the discipline, harmony, and general good of the naval service." We beg leave to ask, who is injuring the "discipline and harmony of the service," when these gentlemen pronounce a regulation of the Hon. Secretary's illegal, and refuse to obey it? Who is impairing the discipline and harmony of the service, when post captains and others indignantly address the navy department, and ask permission to lay aside epaulets, because Surgeons and Pursers have been directed to wear them? Who is destroying the discipline and harmony of the service when officers, commanding on foreign stations, refuse to enforce the general orders transmitted to them, because they conflict with usage and prejudice? The answer is plain: the example of insubordination is set by *superiors* to every stripling in the service.

When an order, issued by the Secretary of the Navy to medical officers in 1848, directing them to inspect, from time to time, the provisions, water, spirits, and general condition of the ship in which they might be serving, with reference to their salubrity, reached a commander in the Gulf of Mexico, (his name is found in the list above enumerated,) he said to the first lieutenant, "If we are not very careful these doctors will take the command from us soon." Are not these the infirm suspicions of derangement? Is a commander in such a frame of mind likely to sustain the "discipline and harmony of the service?"

The following paragraph may well be transcribed here for its pertinence.

"The last number of the London Medical Times, in an article on the reappearance of the scurvy, and alluding to its having been on board the Raritan, Potomac, and Falmouth while operating in the Gulf, says, 'the American nation should demand the dismissal of the medical staff connected with our naval service.'"—*New York Morning Express.*

How, it may be asked, is our naval service, but more especially the medical profession of this country, to escape the odium of such imputations, when the most ordinary prophylactic measures are defeated by the vigilant jealousies of executive officers?

Are not these gentlemen fighting against an apparition conjured by themselves? We repeat, *It is not executive, but assimilated rank* that Surgeons and Pursers desire, and their opponents *know it.* They do not wish to be indebted for the uncertain substitute of "courtesy" so patronisingly offered to them, a species of alms which is ever found to be somewhat barometrical in its range. If these combatant gentlemen fear innovation, if they fear the weakening of their long and ably exercised military authority, is language so meagre that it will be difficult to frame an enactment which shall preclude all danger of such a consequence?

A word in regard to a certain pamphlet entitled—"A few thoughts upon Rank in the Navy." There is little reason to doubt that it was written outside of the service, for, to the honor of the naval service be it still said, that it were not easy to find an individual in it, with sufficient wit and malevolence combined, to produce such a tirade of sophistry and ill nature. Is it a saving clause, however, that these gentlemen have obtained and published it for unjust party purposes?

We sometimes hear Surgeons and Pursers called non-combatants, in depreciation of their claims to equal honors with their brother officers. The annals of the British naval service will show that they have never failed to distinguish themselves whenever the opportunity offered, and any one who cares to enquire, will find that, in our own naval service, the elder Hamilton and the late Purser Breese were distinguished and complimented for their personal intrepidity on Lake Erie. And, were it possible, how gladly would the Surgeon turn from his soul-harrowing occupation below, to avenge the sufferings of his mutilated, dying shipmates.

We have reached the application of this subject. Society generally makes a correct estimate of the relative value of the services of its members. Let these gentlemen open their eyes in time. They have complained, ere now, that Surgeons and Pursers are overpaid. But Surgeons and Pursers will yet attain to *all* they merit, abstractedly and *socially;* and, after all, the sick pillow can but be softer for the *cordial* sympathy of the medical attendant whose mere prescription is not all that is required on ship-board; and the heart can only be lighter under many difficulties for the *friendly advances* of the paymaster.

Much more might be said, but we refrain. A board composed of three post captains, *who are known to be inimical to the establishment of assimilated rank*, has been ordered to convene for the purpose of determining the merits of this question, and to confer with an army board detailed for a like purpose. We conjure these gentlemen, then, to act wisely, and to recommend a definite scale of assimilated rank, based upon just principles; for as surely as they fail to do so, Congress will mend their work.

